# The association of race with thyroid eye disease presentation and outcomes

**DOI:** 10.3389/fopht.2023.1309850

**Published:** 2024-01-23

**Authors:** Diane Wang, Charlotte Marous, Pelin Celiker, Wenyu Deng, Eva Kristoferson, Ali Elsayed, Roman Shinder, Nickisa Hodgson

**Affiliations:** ^1^ Department of Ophthalmology, SUNY Downstate Medical Center, Brooklyn, NY, United States; ^2^ Department of Ophthalmology, Kings County Medical Center, Brooklyn, NY, United States; ^3^ Department of Ophthalmology, West Virginia University, Morgantown, WV, United States; ^4^ Oculoplastic and Orbital Surgery, Wills Eye Hospital, Thomas Jefferson University Hospital, Philadelphia, PA, United States; ^5^ Department of Otolaryngology, SUNY Downstate Medical Center, Brooklyn, NY, United States

**Keywords:** thyroid eye disease (TED), orbit, teprotumumab, EUGOGO, Graves’ disease

## Abstract

**Introduction:**

Classification of thyroid eye disease (TED) is largely based on guidelines developed in Europe and North America. Few studies have investigated the presentation and treatment of TED in Black populations. The objective is to examine the manifestations of TED in secondary and tertiary care center-based populations with a significant proportion of Black patients.

**Materials and methods:**

Retrospective chart review identifying patients with a reported race/ethnicity and a presenting clinical diagnosis of TED at Kings County Hospital and SUNY Downstate Medical Center and affiliated clinics from January 1, 2010 through July 31, 2021. Main outcome measures include age of disease onset, sex, smoking status, insurance status, postal code of residence, clinical exam features, number of follow-up visits, length of follow-up, and treatments received.

**Results:**

Of the 80 patients analyzed, 49 were Black (61.2%) and 31 were White (38.8%). Between Black and White patients, there were differences in the mean age of presentation (48.1 [range 21-76] vs 56.8 [range 28-87] years, P=0.03), insurance status (51.0% vs 77.4% private insurance, P=0.02), and mean follow up length among those with multiple visits (21.6 [range 2-88] vs 9.7 [range 1-48] months, P=0.02). The distribution of EUGOGO scores were not significantly different between Black and White patients. On initial presentation, fewer Black patients had chemosis (OR 0.21, 95% CI, 0.08 to 0.57, P=0.002), and caruncular swelling (OR 0.19, 95% CI, 0.06 to 0.59, P=0.002) compared to White patients. During the overall disease course, fewer Black patients had subjective diplopia (OR 0.20, 95% CI, 0.07 to 0.56, P=0.002), chemosis (OR 0.24, 95% CI, 0.09 to 0.63, P=0.004), and caruncular swelling (OR 0.18, 95% CI, 0.07 to 0.51, P=0.001) compared to White patients. Black patients received oral steroids (42.9% vs 67.7%, P=0.03), intravenous steroids (18.4% vs 16.1%, P=0.8), orbital decompression surgery (16.7% vs 6.5%, P=0.19), and teprotumumab (22.9% vs 22.6%, P=0.99) at similar rates.

**Discussion:**

Black patients presented with fewer external exam findings suggestive of active TED compared to White patients, but the rate of compressive optic neuropathy and decompression surgery were similar in the two groups. These differences may be due to disease phenotypes, which warrant further study.

## Introduction

Thyroid eye disease (TED) is an inflammatory disease in which autoimmune antibodies stimulate orbital fibroblast proliferation, leading to expansion and hypertrophy of orbital tissues. It is the most common autoimmune disease of the orbit, and the most common extrathyroidal manifestation of Graves’ disease (1–3). Though the disease is self-limiting, it continues to be challenging to manage, and severe forms may result in devastating disfigurement and visual impairment. A challenge in managing TED is the variability in disease presentation and treatment response. Many risk factors for the development of severe disease have been studied (4), including genetic predispositions, environmental and lifestyle influences, and anatomical factors (5). However, there is a lack of data on the presentation and treatment of TED in the Black population. Furthermore, the classification of TED has largely been based on guidelines developed in Europe and North America.

Several studies have already revealed differences in TED manifestations between racial populations. For example, TED is commonly associated with upper eyelid retraction; however, Lim et al. showed that lower lid retraction was commonly seen in Chinese, Malay, and East Indian patients (6). A study in India showed that Indian patients with TED had less proptosis than patients of other backgrounds (7). Furthermore, an investigation at a hospital in Accra, Ghana showed that the ocular presentation of patients with TED seemed milder, with fewer cases of severe proptosis or optic neuropathy, than in Caucasians (8).

In addition to affecting disease presentation, racial and socioeconomic factors may also influence treatment decisions and disease outcomes. With the availability of newer treatments such as teprotumumab for TED, further studies of treatment decisions for different patient populations are warranted (9, 10). One must consider not only the clinical manifestations leading to treatment decisions for these patients, but also numerous social determinants of health such as employment status, income, race or access to follow-up care as well as the relationship of these factors with mental health in patients with TED (11, 12). Ultimately, a better understanding of how TED may affect patients of with different socioeconomic backgrounds and hence determinants of health can help us to provide improved care.

To our knowledge, there have been no studies investigating the manifestations of TED or outcomes in the Black population in the United States. Our study seeks to address this gap and better elucidate the association between TED and race by comparatively studying disease prevalence, clinical presentation, and treatment through an analysis of patients seen at Kings County Hospital (KCH) and SUNY Downstate Medical Center (DMC), two major hospitals serving a predominantly Black neighborhood in Brooklyn, New York. Herein, we evaluate a cohort of patients at KCH and DMC over an eleven-year period with the goal of identifying the clinical manifestations, potential risk factors associated with disease prognosis, and treatment approaches.

## Materials and methods

### Study population

This retrospective, non-interventional study included all patients treated in the ophthalmology department of KCH and DMC and its satellite clinics between January 1, 2010 and July 31, 2021 with the diagnosis of thyroid eye disease. This study has been approved by the institutional review board at KCH and DMC. This study followed the Strengthening the Reporting of Observational Studies in Epidemiology (STROBE) reporting guidelines.

The cohort included patients with a diagnosis of TED. A search of the electronic medical record (EMR) system was used to identify all patient records with the following ICD codes: E05.00 (thyrotoxicosis without thyroid storm), E05.01 (thyrotoxicosis with thyroid storm), H05.20 (exophthalmos), H02.539 (eyelid retraction). An additional search of the ophthalmology department’s database of previous consult cases was performed to include all patients with diagnoses including “thyroid eye disease”, “thyroid ophthalmopathy”, and “Graves’ disease”. Race was self-designated by the patient on the EMR.

Data was obtained via chart review to include subjects who fit the following inclusion criteria: patients older than 18 years of age of any gender or sex, a diagnosis of TED within their lifetime, and the presence of ocular symptoms or signs of TED. Patients were excluded if they did not have a self-identified race in their EMR and if they had any other alternative cause of orbital inflammation.

### Clinical data and outcome variables

Recorded patient characteristics include age, sex, smoking status, insurance status, postal code of residence, history of Graves’ disease, history of previous endocrine monitoring, biochemical thyroid status (based on thyroid hormone levels), thyroid stimulating immunoglobulin (TSI) levels, systemic symptoms of hyperthyroidism (*e.g.*, tachycardia, sweating), total number of ophthalmology office visits, and total length of time of follow up in months. EUGOGO grading at the initial visit was collected. The presence of the following exam findings was recorded at the presenting visit: afferent pupillary defect, extraocular movement limitation, pain with extraocular movements, subjective diplopia, eyelid erythema, eyelid edema, eyelid retraction, conjunctival injection, chemosis, caruncular swelling, exophthalmos, exposure keratopathy, and compressive optic neuropathy. Similar data was collected for subsequent visits at 0-3 month, 3-6 month, 6-12 month, and 12-24 month intervals. The treatment modalities implemented, such as oral or intravenous corticosteroids, orbital radiation therapy, teprotumumab vs other immunomodulatory therapies, and decompression surgery were recorded.

### Data analysis

The data was analyzed using SPSS (Version 28.0.1.0, IBM Corp, 2021). Demographic characteristics and the presence of exam findings were compared between groups using Mann-Whitney U tests and 2-tailed *t* tests for continuous variables and Chi-square tests for discrete variables. Multivariate logistic regression with age, sex, race, smoking status, and insurance status as covariates were used to assess factors associated with the presence of clinical exam findings. P<0.05 was used as the threshold of significance for all analyses, with a Bonferroni correction implemented in cases of multiple-category comparisons.

## Results

A total of 91 patients presented to KCH and DMC with a diagnosis of thyroid eye disease during the eleven year study period. Race included Black (n=49, 53.8%), White (n=31, 34.1%), Asian (n=5, 5.5%), non-Black Hispanic (n=3, 3.3%), other (n=3, 3.3%). Due to a small sample size, the 11 patients of other races (5 Asian, 3 Hispanic, 3 other) were excluded. This yielded a final study population of 80 patients, including 49 Black patients (61.2%) and 31 White patients (38.8%). **Table 1** presents patient baseline characteristics, including demographics and prior thyroid disease monitoring.

**Table 1:** Patient Baseline Characteristics.

	No (%)			
Characteristic	Overall	Black	White	p value
**Number**	80	49	31	
**Age, mean (SD), years**	51.5 (16.4)	48.1 (16.6)	56.8 (15.0)	**0.03**
Sex				
Female	63 (78.8)	38 (77.6)	25 (80.6)	
Male	17 (21.2)	11 (22.4)	6 (19.4)	
Current Smoker				
Yes	20 (25.0)	9 (18.4)	11 (35.5)	0.08
No	60 (75.0)	40 (82.6)	20 (64.5)	
Insurance				
Uninsured or Medicaid	28 (35.0)	22 (44.9)	6 (19.4)	**0.02**
Private Insurance	49 (61.3)	25 (51.0)	24 (77.4)	
Not reported	3 (3.8)	2 (4.1)	1 (3.2)	
**Median Income Based On Postal Code of Residence^1^**	$59,655	$53,559	$69,291	0.002
< $40,000	14 (17.5)	13 (26.5)	1 (3.2)	
$40,000 - $80,000	51 (63.8)	30 (61.2)	21 (67.7)	
> $80,000	15 (18.8)	6 (12.2)	9 (29.0)	
Previous Diagnosis of Graves’ disease				
Yes	59 (73.8)	40 (81.6)	19 (61.3)	0.04
No	21 (26.2)	9 (18.4)	12 (38.7)	
Previous Endocrine Monitoring				
Yes	65 (81.3)	41 (83.7)	24 (77.4)	0.49
No	15 (18.7)	8 (16.3)	7 (22.6)	

^1^American Community Survey. Median Income In The Past 12 Months (In 2019 Inflation-Adjusted Dollars), Table S1903. Washington, D.C.: U.S. Census Bureau; 2019. Reaches significance.

**Figure 1:**
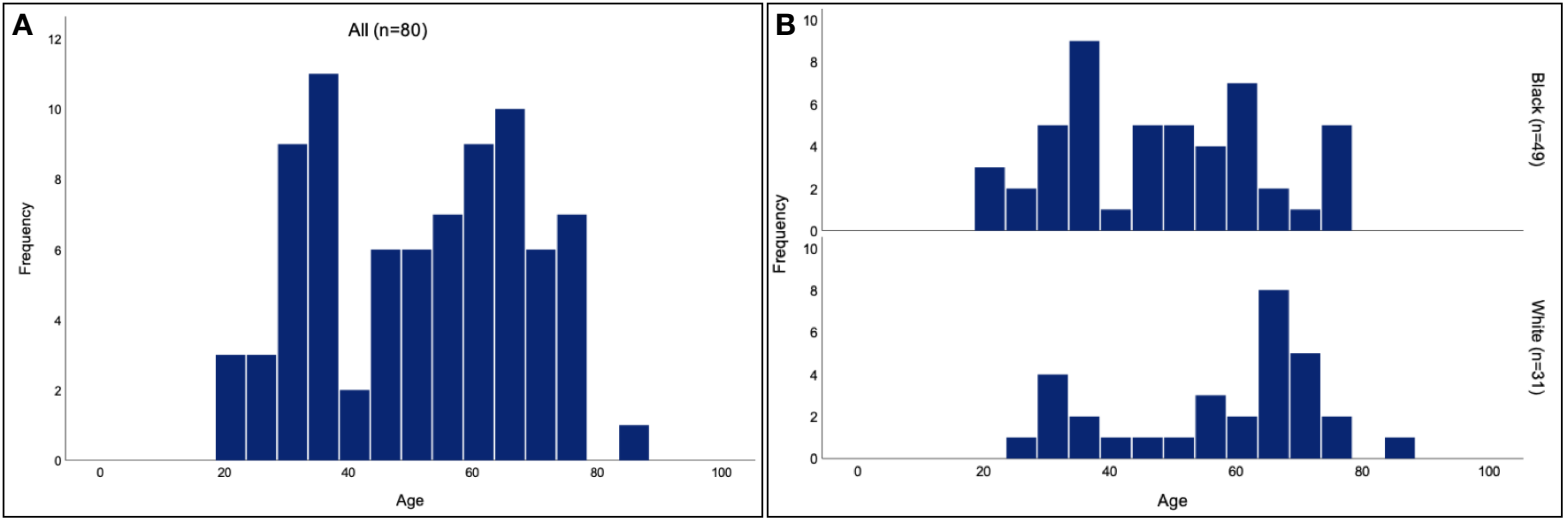
Age of TED presentation for Black and White patients. Histogram of the age of presentation for all patients **(A)** shows a bimodal distribution, with a mean age of 51.5 years. Separate histograms for the age of presentation in Black and White patients **(B)** similarly show bimodal distributions, with a mean age of 48.1 years for Black patients and 56.8 years for White patients.

Of the 80 patients analyzed, 63 (78.8%) were female and 17 (21.2%) were male. The overall mean age of presentation was 51.5 years (range 21-87 years) and the age of presentation followed a bimodal distribution. The mean age of presentation was lower for Black patients (48.1 years, range 21-76 years) than for White patients (56.8 years, range 28-87 years). Both groups maintained a bimodal age distribution (**Figure 1**). There were 11 (35.5%) White patients and 9 (18.4%) Black patients who were active smokers (P=0.08).

Of the 47 Black patients and 30 White patients whose insurance status was reported, 22 (46.8%) Black patients and 6 (20.0%) White patients were uninsured or on Medicaid (P=0.02).

There was no significant difference between Black patients and White patients in the rates of previous Graves’ disease diagnosis (81.6% vs 61.3%, P=0.04) and previous endocrine monitoring (83.7% vs 77.4%, P=0.49), with a significance level set at p<0.025 after Bonferroni correction. Of those who had available laboratory results, both Black and White groups were similar in the number of biochemically hyperthyroid individuals (62% [26/42] vs 67% [12/18], P=0.73) based on serum TSH levels within 6 months of their presentation for TED. 27% of Black patients and 45% of White patients had prior treatment with oral steroids (p=0.09).

**Figure 2:**
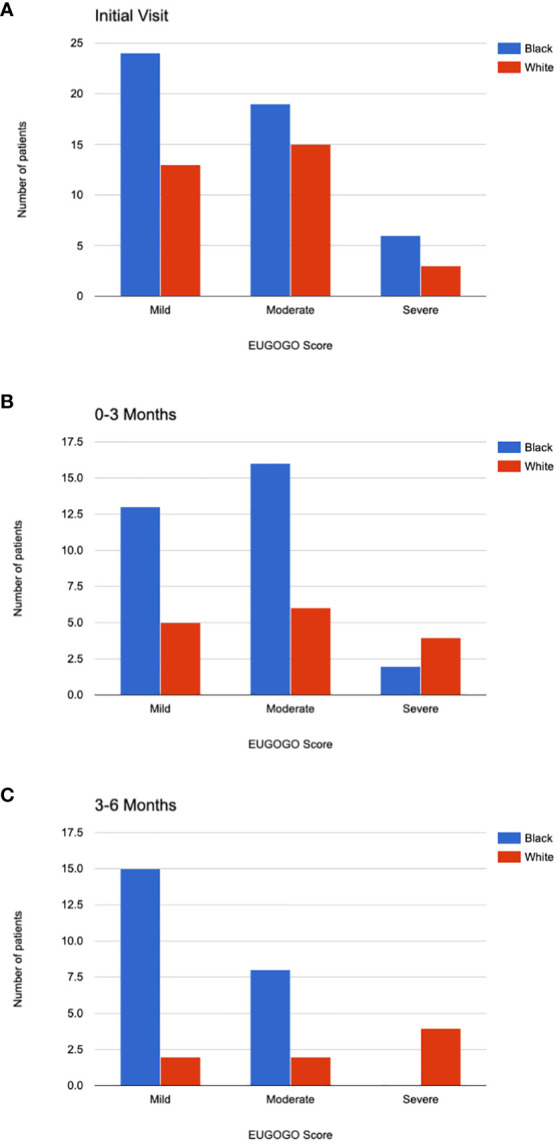
EUGOGO Scores. EUGOGO scores for Black and White patients are shown at their initial visit **(A)**. Scores are shown for those who followed up 0-3 months after the initial visit **(B)**, 3-6 months after the initial visit **(C)**. The differences in EUGOGO scores between the racial groups were not significantly different after Bonferroni correction for multiple comparisons.

The EUGOGO scores were compared between Black patients and White patients (**Figures 2A-C**). There was no significant difference in the distribution of mild, moderate, or severe disease between the two groups at the initial visit (p=0.72) and among those who followed up at 0-3 months (p=0.28) and 3-6 months (p=0.02). The significance level was set at p<0.17 after Bonferroni correction. Due to the fewer number patients who attended follow up at 6-12 months and 12-24 months after the initial visit, comparison of disease severity was not compared for those time periods.

A comparative analysis of exam findings at initial visit and overall is shown in **Table 2**. The significance level was set at p<0.004 after Bonferroni correction. The most common sign of TED in Black patients overall was proptosis (87.8%), while the most common sign in White patients overall was eyelid retraction (90.3%). On initial presentation, there was no significant difference in the rate of EOM limitation (44.9% vs 58.1% P=0.33) or subjective diplopia (34.7% vs 67.7%, P=0.004). Significantly fewer Black patients had chemosis (OR 0.21, 95% CI, 0.08 to 0.57) and caruncular swelling (OR 0.19, 95% CI, 0.06 to 0.59) compared to White patients. Overall, at any time during their follow-up, significantly fewer Black patients had diplopia (OR 0.20, 95% CI, 0.07 to 0.56), chemosis (OR 0.24, 95% CI, 0.09 to 0.63), and caruncular swelling (OR 0.18, 95% CI, 0.07 to 0.51).

**Table 2:** TED examination features at initial visit and overall disease course for Black and White patients.

	Initial Visit				Overall			
Exam Features	Black, No. (%)	White, No. (%)	p-value^a^	Odds Ratio (95% CI)	Black, No. (%)	White, No. (%)	p-value^a^	Odds ratio (95% CI)
Afferent pupillary defect	4 (8.2)	0 (0)			7 (14.2)	5 (6.5)	0.82	
Extraocular movement limitation	22 (44.9)	18 (58.1)	0.25		29 (59.2)	21 (67.7)	0.44	
Pain with extraocular movements	11 (22.4)	10 (32.3)	0.33		16 (32.7)	16 (45.2)	0.09	
Subjective diplopia	17 (34.7)	21 (67.7)	0.004		20 (40.8)	24 (77.4)	0.002	0.2 (0.07, 0.56)
Proptosis	39 (79.6)	24 (77.4)	0.82		43 (87.8)	26 (83.9)	0.62	
Eyelid retraction	38 (77.6)	26 (83.9)	0.49		41 (83.7)	28 (90.3)	0.40	
Lagophthalmos	25 (51.0)	15 (48.4)	0.82		26 (53.1)	17 (54.8)	0.88	
Eyelid edema	18 (36.7)	17 (54.8)	0.11		24 (49.0)	20 (64.5)	0.17	
Conjunctival injection	22 (44.9)	20 (64.5)	0.09		28 (57.1)	23 (74.2)	0.12	
Chemosis	10 (20.4)	17 (54.8)	0.002	0.21 (0.08, 0.57)	15 (30.6)	20 (64.5)	0.003	0.24 (0.09, 0.63)
Exposure keratopathy	5 (10.2)	3 (9.7)	0.94		7 (14.3)	3 (9.7)	0.54	
Caruncular swelling	6 (12.2)	13 (41.9)	0.002	0.19 (0.06, 0.59)	8 (16.3)	16 (51.6)	0.001	0.18 (0.07, 0.51)
Compressive optic neuropathy	8 (16.3)	2 (6.5)	0.19		11 (22.4)	3 (9.7)	0.14	

a Significance level set at p<0.004 after Bonferroni correction.

Multivariate logistic regression analysis again demonstrated significant associations between White race and subjective diplopia (OR 3.86, 95% CI 1.1 to 13.2, P = 0.03), chemosis (OR 3.5, 95% CI 1.2 to 10.2, P=0.02), and caruncular swelling (OR 4.3, 95% CI 1.3 to 14.1, P=0.02). Additionally, an age greater than 65 years was associated with a 7-fold likelihood of having extraocular movement limitation (95% CI 1.2 to 42.1, P=0.03).

The mean follow up time for all patients was 12.0 months (range 0-88 months), with an average of 5.3 total visits (range 1-56 visits). Black patients followed up for longer (15.9 months [range 0-88] vs 5.9 months [range 0-48], p=0.01). However, the total number of number of visits attended by Black and White patients was not significantly different (6.5 visits vs 3.3 visits, p=0.046), with a significance level set at p<0.025 after Bonferroni correction.

A comparative analysis with Bonferroni correction revealed no significant difference in the rate of treatment with oral steroids (22.4% vs 48.4%), intravenous steroids (18.4% vs 16.1%), thyroidectomy (20.8% vs 19.4%), orbital radiation therapy (4.2% vs 3.2%), teprotumumab (22.9% vs 22.6%), or decompression surgery (16.7% vs 6.5%) between racial subgroups.

## Discussion

This retrospective study of patients treated in Kings County, Brooklyn, New York over 11 years identified 91 patients who presented for TED, 49 of whom were Black and 31 of whom were White. To our knowledge, this is the first study to evaluate manifestations of TED in a population of primarily Black patients in the United States.

Our study found the mean age of TED presentation to be younger in Black patients than in White patients, though both populations showed a bimodal distribution with peaks at around 30 years and 65 years of age. In both groups, the female to male ratio was roughly 4:1, consistent with prior epidemiologic studies (13, 14). Though there was not a significant difference between the two groups in smoking status, the p value of 0.08 approaches significance in a small patient population and may potentially be a confounding factor. The distribution of EUGOGO scores were not significantly different between Black and White patients. Of the exam findings that were assessed in our chart review, Black patients on average had less subjective diplopia, chemosis, and caruncular injection than White patients. Despite this, Black patients did not appear to have better outcomes. The rate of compressive optic neuropathy was similar between Black and White patients. The two groups had similar rates of patients who received steroids, teprotumumab, and decompression surgery.

Manifestations of TED in Black patients may differ from White patients and the metrics used in our study to define TED may not fully account for how the disease presents in Black populations. The exam findings included our study are derived from epidemiologic studies and guidelines in Europe and North America (2, 13, 15–21). These guidelines, based on a predominantly Caucasian population, are currently widely used to assess and monitor TED. In 1969, Dr. Werner from the New York Presbyterian Hospital of New York, described the NO SPECS system to classify TED (20, 21). To better classify active and non-active disease, the clinical activity score (CAS) system was developed in 1999 in Amsterdam (18), and was amended by the European Group on Graves’ Orbitopathy (EUGOGO) (2, 15, 16). Though the original studies did not specify the racial demographics of the included patients, a later analysis showed that over 95% of the TED patients referred in the EUGOGO centers were Caucasian (22). The VISA classification was developed in 2006 in British Columbia, and assesses four categories: V (vision); I (inflammation/congestion); S (strabismus/motility restriction); and A (appearance/exposure) (17). Currently, the EUGOGO classification system is used more commonly in Europe, while the VISA classification system is used more commonly in the United States and Canada (23). A frequently-cited epidemiologic study of TED in the United States was based on a 1994 cohort in Olmsted County, Minnesota. In that cohort, 100% of the patients were White (12).

There have been several studies on the development, manifestations, and outcomes of TED and Graves’ disease in Black populations. A study of 75 patients in Nigeria showed that the rate of severe ocular complications is low, with the painless eye swelling as the most common symptom (67%). Chemosis and diplopia were both rare (5%) (24). A study of 194 patents with thyroid disorders in Ghana found, similarly, that TED presentation tended to be milder than in Caucasians (8). Other studies comparing TED in Black and White patients did not show differences in disease incidence and severity between races; however most of these studies comprised largely of Caucasian patients. Stein et al. found no association between race and the development of TED in 8404 patients with Graves’ disease, though only 7% of the cohort was classified as being of Black race (25). Edmunds et al. found that lower socioeconomic status but not race, was associated with severe TED (26). Their cohort consisted of 77% White patients and 11% Black patients in the United Kingdom, and social grade was based upon income, employment, education level, disability, and access to care.

Black patients in this study presented with what appeared as a more indolent presentation of TED. However, the rates of compressive optic neuropathy were similar in the two races, as was the rate of surgical decompression, treatment with intravenous steroids, and treatment with teprotumumab. The current guidelines for classifying TED may not encompass the disease manifestations that occur in Black patients. For example, the Ghana study by Ackuaku et al. showed that a quarter of patients with TED had glabellar furrows and 41% had increased intraocular pressure on upgaze (8), both of which are clinical features not traditionally used in the grading of TED. Studies of TED in other races also found clinical manifestations in those populations that are not documented in Caucasian studies. Lim et al. proposed that lower lid retraction be included as a diagnostic criterion for TED for Chinese, Malay, and Indian Asians, as it was present in 60% of cases (6). Similarly, Black patients may have fewer external inflammatory findings or may have other clinical findings that are not accounted for in current clinical criteria.

There are several limitations to this study. It is retrospective in nature, with a relatively small sample size. The EMR at two of the clinical sites were started in 2017, so ICD searches may have missed patients with visits prior to that year. The exact timing from the onset of symptoms to initial visit was not available in every patient chart. Therefore, we were not able to assess symptom chronicity as a possible contributing factor for the differences in clinical exam findings between Black and White patients. Additionally, we had limited data to conclude the reasons for the difference in follow-up time between the two groups, such as discharge from clinic or loss to follow up. Lastly, though a thorough chart review was conducted to collect clinical data, we were not able to use CAS or VISA score as a metric of disease severity due to lack of complete and consistent documentation using those guidelines. For example, CAS criteria such as “spontaneous orbital pain” were not always documented, nor were exact Hertel measurements to determine the increase in proptosis from one visit to the next. Specific grading scores of findings as indicated on the VISA guidelines were also not used in our patient charts. A prospective study including more quantifiable measures of clinical findings would be useful.

This is the first study that compares TED manifestations and outcomes between Black and White patients in a primarily Black population. In our study, Black patients with TED presented with fewer clinical exam features based on current classification guidelines, but had longer follow-up and underwent similar treatments as White patients. This may suggest there are additional factors influencing how the disease manifests and is treated in Black and White patients. Further studies are needed to explore these factors and determine whether the differences are related to biologic, socioeconomic, or other differences between the racial groups.

## Data availability statement

The original contributions presented in the study are included in the article/supplementary material. Further inquiries can be directed to the corresponding author.

## Ethics statement

The studies involving humans were approved by institutional review board at Kings County Hospital (KCH) and SUNY Downstate Medical Center (DMC). The studies were conducted in accordance with the local legislation and institutional requirements. Written informed consent for participation was not required from the participants or the participants’ legal guardians/next of kin in accordance with the national legislation and institutional requirements.

## Author contributions

DW: Writing – original draft, Writing – review & editing. CM: Writing – original draft. PC: Writing – original draft, Writing – review & editing. WD: Writing – original draft. EK: Writing – review & editing. AE: Data curation. RS: Writing – original draft. NH: Writing – original draft.
